# Lightweight Semantic-Guided FCOS for In-Line Micro-Defect Inspection in Semiconductor Manufacturing

**DOI:** 10.3390/mi17040473

**Published:** 2026-04-14

**Authors:** Tao Zhang, Shichang Yan, Gaoe Qin

**Affiliations:** 1 School of Electronic Information, Central South University, Changsha 410075, China; 2 School of Artificial Intelligence and Computer Science, Jiangnan University, Wuxi 214122, China; 3 Jiangsu Huaying Intelligent Technology Co., Ltd., Wuxi 214101, China; qingaoe@hwaintek.com

**Keywords:** semiconductor manufacturing, automated optical inspection (AOI), micro-defect detection, printed circuit board (PCB), FCOS, edge computing, semantic-guided feature fusion

## Abstract

The relentless miniaturization of semiconductor components and Printed Circuit Boards (PCBs) has rendered Automated Optical Inspection (AOI) of micro-defects a critical bottleneck in modern manufacturing and metrology. While in-line inspection systems offer economically viable and scalable quality control solutions, they impose stringent constraints on both inference latency and detection robustness—particularly for diminutive, sparsely distributed defects (e.g., mouse bites, pinholes) amidst complex, repetitive circuit topologies. To bridge this gap, we present a semantic-enhanced FCOS framework specifically engineered for micro-defect inspection. Our approach introduces two synergistic innovations: (1) a Semantic-Guided Upsampling Unit (SGU) that adaptively reweights channel–spatial features to reconcile the semantic disparity between shallow textural details and deep contextual representations; and (2) a Sparse Center-ness Calibration (SCC) module that enforces high-confidence, spatially sparse supervision to sharpen localization precision and suppress false positives. The SGU is integrated within a Progressive Semantic-Enhanced Feature Pyramid Network (PSE-FPN) that extends multi-scale representations to stride-4 (P2) resolution, while the SCC module is embedded directly into the detection head. Comprehensive evaluations on MS COCO and the real-world DeepPCB dataset validate the efficacy of our design. On COCO, our model achieves 41.8% AP with real-time throughput of 28 FPS on a single NVIDIA 1080Ti GPU. A lightweight variant further attains 41.6% AP at 42 FPS, accommodating high-throughput production environments. For PCB defect detection, the framework delivers 98.7% mAP@0.5, substantially outperforming contemporary detectors. These results demonstrate that semantics-aware, lightweight architectures enable scalable, real-time quality assurance in semiconductor manufacturing.

## 1. Introduction

With the relentless miniaturization of semiconductor components and the increasing complexity of Printed Circuit Boards (PCBs), the industry faces escalating challenges in maintaining high manufacturing yield and product reliability. Among these, micro-defects—such as microscopic scratches, pinholes, open circuits, and mouse bites—have become a persistent issue affecting the functionality and lifespan of electronic devices [1,2,3,4]. Traditional quality control heavily relies on manual inspection or conventional optical methods, which are not only time-consuming but also prone to missing small, sparse defects due to operator fatigue and the sheer volume of production. As semiconductor manufacturing shifts toward Industry 4.0 and intelligent paradigms, there is an urgent need for automated, scalable, and fine-grained monitoring solutions [5,6].

Recent advances in Automated Optical Inspection (AOI) equipment have created unprecedented opportunities for ubiquitous quality monitoring across the production line. These systems continuously generate high-resolution visual data, forming a distributed perception network that can support real-time metrology and defect detection. When coupled with artificial intelligence (AI), such systems enable edge-intelligent analysis directly on resource-constrained platforms (e.g., portable or edge-deployed AOI nodes), paving the way for responsive and sustainable quality assurance in the semiconductor industry.

However, detecting micro-defects—often smaller than 32 × 32 pixels—against the cluttered and highly repetitive background of circuit patterns remains highly challenging. While modern object detectors have achieved remarkable success on benchmark datasets like COCO, they are primarily optimized for medium-to-large objects under balanced distributions [7,8]. When applied to extreme-scale, sparse instances in real-world AOI systems, two critical limitations emerge:

(1) Semantic misalignment in feature fusion: Shallow features rich in texture details suffer from semantic noise (often exacerbated by complex circuit traces), while deep features lack spatial fidelity; conventional upsampling methods (e.g., bilinear interpolation) fail to bridge this “semantic gap”, leading to degraded localization accuracy for tiny defects [9,10,11,12,13,14].
(2) Ineffective quality estimation: Standard center-ness learning in anchor-free frameworks assigns supervision signals uniformly across all positive samples within ground-truth boxes, diluting focus on central regions and allowing low-quality predictions near boundaries (e.g., confusing a defect edge with a normal circuit line) to survive non-maximum suppression (NMS) [15,16,17].

To address these issues, we propose a semantic-enhanced FCOS framework specifically designed for micro-defect detection in semiconductor manufacturing. Our approach introduces two core innovations: (i) A Semantic-Guided Upsampling Unit (SGU) that dynamically modulates low-level features through channel–spatial reweighting, ensuring semantically coherent feature propagation from deep to shallow layers. (ii) A Sparse Center-ness Calibration (SCC) module that enforces sparse, high-confidence supervision only on well-aligned predictions, thereby enhancing spatial focus and suppressing false positives. Specifically, the SGU is integrated into a Progressive Semantic-Enhanced Feature Pyramid Network (PSE-FPN), which extends multi-scale representation down to P2-level resolution (stride = 4) to preserve spatial details, while the SCC module is embedded within the detection head to refine localization quality.

We evaluate our method on both the MS COCO benchmark and the real-world DeepPCB dataset for PCB defect detection. Experimental results show that our framework achieves 41.8% AP on COCO, significantly outperforming the baseline FCOS and contemporary state-of-the-art anchor-free methods. It maintains a real-time performance of 28 FPS on a single NVIDIA 1080Ti GPU, with our lightweight variant further reaching 42 FPS while preserving a high accuracy of 41.6% AP. These results demonstrate strong practicality for deployment on industrial Automated Optical Inspection (AOI) systems. Furthermore, application validation on the DeepPCB dataset yields a superior mAP@0.5 of 98.7%, confirming the effectiveness of our approach in mitigating background interference and improving small-defect sensitivity in real-world semiconductor metrology scenarios.

## 2. Related Work

### 2.1. Deep Learning-Based Object Detection Methods

Modern object detection frameworks can be broadly categorized into two paradigms based on whether they rely on pre-defined anchor boxes: anchor-based and anchor-free. Anchor-based methods, such as Faster R-CNN [18], SSD [19], and RetinaNet [20], achieve stable performance on medium-to-large-scale objects by matching ground-truth instances with densely sampled anchors. However, these approaches suffer from redundant anchor designs, sensitivity to hyperparameters (e.g., aspect ratios and scales), and high computational overhead—challenges that become particularly pronounced in small-object detection and edge deployment scenarios.

In contrast, anchor-free detectors predict bounding boxes directly from feature map pixels without requiring IoU-based matching, resulting in simpler architectures and reduced inference latency. This property has made them increasingly popular for lightweight and real-time applications on resource-constrained platforms—a key requirement for scalable Automated Optical Inspection (AOI) systems and edge-computing nodes in semiconductor manufacturing. Recent advances include YOLOv12 [21], YOLO-NAS [22], and RF-DETR [23], which leverage techniques such as region-wise attention, neural architecture search (NAS), or depth-wise separable convolutions to achieve real-time performance on general benchmarks like COCO. Despite their efficiency, most of these models are optimized for average-scale object detection; excessive downsampling in deep layers leads to early loss of fine-grained texture details, significantly degrading recall rates for tiny objects. Transformer-based variants eliminate post-processing steps but often employ heavy backbones with high memory and computation costs, making them less suitable for edge devices. Moreover, extremely compressed convolutional channels reduce discriminative power under sparse activations, while global attention mechanisms tend to average localization errors across dense predictions—limiting generalization to new environments and increasing retraining costs.

To address the unique challenges of PCB and semiconductor inspection, several domain-specific models have recently been proposed, predominantly building upon anchor-free architectures. For instance, Su et al. [24] proposed TCPNet, which utilizes a twin-connected parallel network and a Differential Feature Remodeling Module (DFRM) to extract semantic and texture information from local to global scales. Similarly, Ou and Wang [25] developed YOLO-DTS, which utilizes a Dual-Transformer downsampling module and deformable attention within the YOLOv8 framework to adapt to irregular defect morphologies. In a different vein, to reduce reliance on large annotated datasets, Yin et al. [26] proposed a zero-shot detection framework that formulates the problem as a pixel-level segmentation task, simulating image differencing guided by optical flow and reference reconstruction. While these specialized methods achieve impressive accuracy on specific benchmarks, they often rely on computationally expensive Transformer blocks, deformable convolutions, or intensive image-pair alignments. These heavy components significantly increase the computational burden and memory footprint, which can hinder real-time deployment on resource-constrained edge AOI equipment.

Among anchor-free frameworks, FCOS [27] stands out due to its fully convolutional design, multi-scale feature pyramid (FPN) [28], and center-ness branch for suppressing low-quality proposals. By preserving high-resolution features and avoiding hand-crafted anchor priors, FCOS offers a favorable trade-off between accuracy and simplicity, making it particularly suitable for detecting tiny, complex defects on high-density circuit boards in low-compute edge-inspection systems—such as those deployed on portable or edge-node AOI equipment.

### 2.2. FCOS-Based Detection Frameworks

Since its introduction, FCOS has inspired numerous extensions aimed at improving detection performance while maintaining deployability on edge platforms. These efforts fall into two main directions: backbone-lightening with feature enhancement, and head-level structural refinement.

Firstly, Chen et al. [29] replaced the standard ResNet backbone with MobileNetV3 [30], leveraging inverted residuals and depth-wise convolutions to reduce model size. They further introduced the ECA attention module [31] and a bidirectional feature pyramid (DEFPN) to enhance representation for occluded and small targets. Yang et al. [32] proposed an Adaptive Spatial Feature Fusion (ASFF) module that dynamically learns fusion weights across scales via attention, enabling the network to focus more on informative regions for small objects. Li et al. [33] designed a Learnable Upsampling FPN (L-UFPN), replacing conventional interpolation with learnable deconvolution kernels to better preserve spatial details during upsampling. Luo et al. [34] combined the dense prediction philosophy of FCOS with Transformer-like long-range modeling through State Space Models (SSMs), introducing an Internal Feature Enhancement Module (IFEM) to capture contextual dependencies in crowded scenes.

The second line of research focuses on refining the tri-head prediction structure (classification, regression, and center-ness) of FCOS: Zhang et al. [35] addressed semantic dilution in deep layers and inconsistency between regression and center-ness branches by proposing a Multi-scale Fusion (MF) path and an Interaction Learning (IL) mechanism. The MF adds a bottom-up connection to retain local details, while IL uses a center-ness-weighted mask to recalibrate regression features, promoting cross-branch synergy. Another study by Zhang et al. [36] argued that the core difference between anchor-based and anchor-free methods lies not in regression form but in positive/negative sample assignment. Their Adaptive Training Sample Selection (ATSS) retains FCOS’s three-branch head but introduces dynamic IoU thresholds to select training samples, effectively balancing classification and regression distributions and mitigating the misalignment between predicted quality scores and actual localization accuracy. Li et al. [37] revisited the loss function design, proposing Generalized Focal Loss v2 (GFLv2): The scalar center-ness score is extended to joint quality estimation (IoU + localization confidence), optimized via Quality Focal Loss. Meanwhile, the 4-D regression output is modeled as a probability distribution using Distribution Focal Loss, enabling end-to-end optimization of all three tasks. Qiu et al. [38] observed boundary degradation in regression outputs and added an ultra-lightweight Border Branch alongside the original head. This auxiliary branch extracts deformable convolution features from 1 to pixel-wide strips along each side of the predicted box and produces residual corrections, refining initial offsets in a post-refinement manner.

Despite their advancements, all these works maintain the fundamental “dense prediction + per-pixel center-ness” framework of FCOS. Crucially, the center-ness branch still assigns supervision signals uniformly across all pixels within ground-truth boxes—even those near edges—leading to noisy gradients and poor discrimination between central and marginal predictions. During NMS, this results in the survival of low-quality boxes, especially detrimental for micro-targets where precise spatial focus is essential.

While prior efforts have enhanced either feature semantics or sample selection, the problem of semantic misalignment in shallow-layer fusion and ineffective spatial focusing in quality estimation remains unresolved—particularly critical in micro-defect inspection for semiconductor components and PCBs, where both high-resolution detail preservation and robust false-positive suppression against complex circuit backgrounds are strictly required. Our work addresses this gap by co-designing semantic-guided upscaling and sparse quality calibration within a progressive feature pyramid, enabling accurate and reliable micro-defect detection on edge-ready metrology platforms.

## 3. Methodology

**Figure 1:**
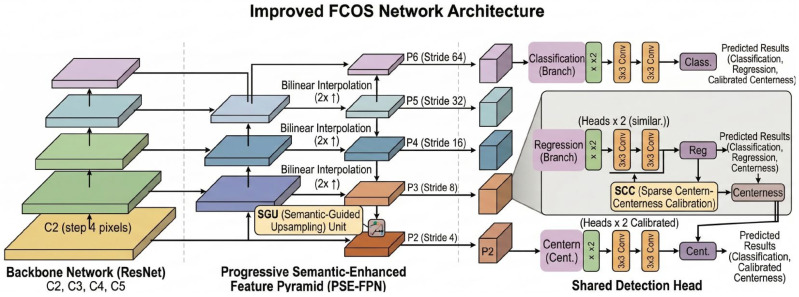
The overall architecture of the proposed semantic-enhanced FCOS framework for micro-defect detection. It consists of a ResNet backbone, a Progressive Semantic-Enhanced FPN (PSE-FPN) integrated with the SGU, and the detection head embedded with the SCC module.

To address the challenges of detecting extremely small and sparse micro-defects—such as mouse bites, pinholes, and open circuits—against complex circuit backgrounds using resource-constrained edge-computing inspection devices, we propose a semantic-enhanced FCOS framework that improves both feature representation and prediction reliability. The overall architecture of the proposed network is illustrated in Figure 1.

Our approach introduces two core components: (i) A Semantic-Guided Upsampling Unit (SGU) to bridge the semantic gap between deep context and shallow details. (ii) A Sparse Center-ness Calibration (SCC) module to enhance spatial focusing in quality estimation. Specifically, the SGU is integrated into a Progressive Semantic-Enhanced Feature Pyramid Network (PSE-FPN), which extends multi-scale feature resolution down to stride-4 while selectively strengthening bottom-up semantic flow, whereas the SCC module is embedded directly into the detection head.

### 3.1. Semantic-Guided Upsampling Unit (SGU)

Traditional upsampling operations—such as bilinear interpolation or transposed convolution—focus primarily on geometric reconstruction and often neglect semantic consistency during feature fusion. To overcome this limitation, we design the SGU unit, inspired by the channel attention mechanism in SENet, but extended in three key aspects:

(1) The calibration branch is relocated from within residual blocks to the skip connection between high-level and low-level features, enabling global semantic priors to directly modulate detailed textures.
(2) The “channel–scalar” reweighting is upgraded to a “channel–spatial” repeated mask, ensuring pixel-wise semantic constraints along the upscaling path.
(3) Sub-pixel convolution (PixelShuffle) replaces global scaling operations, achieving simultaneous enhancement of detail recovery and semantic coherence.

**Figure 2:**
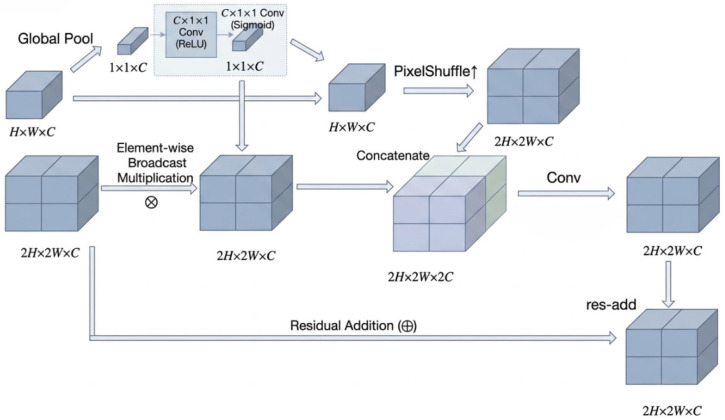
Architecture of the Semantic-Guided Upsampling Unit (SGU). The high-level feature $F_{h}$ generates a channel–spatial attention mask $M_{s}$, which is applied to both $F_{h}$ and $F_{l}$. Sub-pixel convolution ensures detail-preserving upscaling, while residual fusion maintains gradient flow.

The architecture of the proposed SGU unit is illustrated in Figure 2. Given a high-level feature map $F_{h}\in R^{C\times H\times W}$ and a low-resolution feature $F_{l}\in R^{C\times 2H\times 2W}$ to be upsampled, SGU first generates a global semantic prior $M_{s}$ via a lightweight gating network:

(1) $$M_{s}=\sigma \left(W_{2}\cdot \delta \left(W_{1}\cdot \mathrm{GAP}\left(F_{h}\right)\right)\right),$$

where GAP(·) denotes global average pooling, $W_{1}\in R^{C/4\times C}$ and $W_{2}\in R^{C\times C/4}$ are learnable parameters, δ is the ReLU activation, and σ is the Sigmoid function.

The semantically guided upsampling is then defined as follows:

(2) $$F_{h}^{↑}=T\left(F_{h}⊙\mathrm{Repeat}\left(M_{s}\right)\right),$$

where ⊙ denotes element-wise multiplication, Repeat(·) replicates $M_{s}$ across spatial dimensions, and *T* represents the sub-pixel convolution (PixelShuffle) operation.

Finally, the output feature is fused via a residual connection:

(3) $$F_{\mathrm{out}}={\mathrm{Conv}}_{3\times 3}\left(\left[F_{l}⊙\mathrm{Repeat}\left(M_{s}\right),\;F_{h}^{↑}\right]\right)+F_{l},$$

where [·,·] denotes channel concatenation. This formulation ensures that the enhanced semantics from deeper layers guide the restoration of fine-grained details in shallow features, without introducing significant computational overhead.

### 3.2. Progressive Semantic-Enhanced Feature Pyramid Network (PSE-FPN)

The performance of one-stage dense detectors like FCOS heavily relies on the expressive power of the Feature Pyramid Network (FPN). To further improve micro-defect detection accuracy in real-world Automated Optical Inspection (AOI) scenarios, we integrate the SGU unit into FCOS’s FPN architecture and introduce an additional high-resolution level—P2—to strengthen semantic perception in early layers.

**Figure 3:**
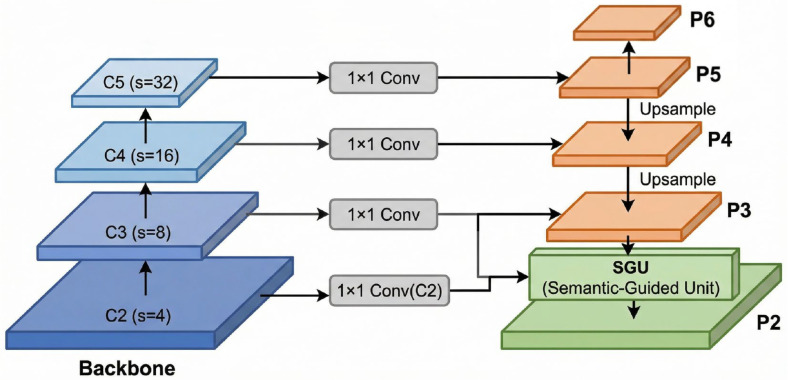
Overview of the Progressive Semantic-Enhanced Feature Pyramid Network (PSE-FPN). Our framework extends FCOS with five-level pyramid {P2–P6}, where only the P3→P2 fusion path employs the proposed SGU unit for semantic-guided enhancement. This selective design strengthens shallow-layer semantics critical for tiny object detection.

As illustrated in Figure 3, PSE-FPN builds upon the original FCOS FPN with two key enhancements:

(1) Hierarchical Extension: We introduce a new feature level P2 derived from the backbone’s C2 output (stride = 4), forming a five-level pyramid {P2,P3,P4,P5,P6}. The high-resolution P2 layer provides essential spatial detail for detecting micro-sized objects commonly found in high-density circuit patterns captured by Automated Optical Inspection equipment.
(2) Selective Enhancement: In the top-down fusion pathway, standard lateral connections with bilinear upsampling are retained for mid-to-high levels (e.g., P4→P3, P5→P4), where deep features already possess strong semantics and alignment precision is more critical. However, for the P3→P2 fusion—the most vulnerable to semantic misalignment due to noisy C2 features—we replace conventional upsampling with the proposed SGU unit.

Formally, given the backbone outputs C2 and C3, we first project C2 to the common channel dimension using a 1×1 convolution:

(4) $$F_{C2}={\mathrm{Conv}}_{1\times 1}\left(C2\right).$$

Then, P3 and $F_{C2}$ are fed into the SGU module to produce the enhanced P2 feature:

(5) $$P2=\mathrm{SGU}(P3,F_{C2}),$$

with SGU following Equations (1)–(3). After obtaining P2, the rest of the pyramid (P3 to P6) is constructed using standard FPN operations.

This selective design is motivated by the observation that the semantic gap between P3 and C2 is the primary bottleneck in preserving contextual integrity for tiny object localization, while higher-level fusions benefit more from geometric refinement than semantic modulation.

### 3.3. Sparse Center-Ness Calibration (SCC)

In FCOS, the center-ness branch suppresses low-quality predictions far from the defect center. However, its ground-truth assignment includes all pixels within the ground-truth box, leading to two critical issues:

(i) Ambiguous supervision: Boundary pixels inherently have lower center-ness values but are still forced to fit non-zero targets, introducing gradient noise.
(ii) Foreground-background imbalance: Approximately 90% of supervisory signals originate from edge or background regions, diluting focus on central areas crucial for precise localization.

**Figure 4:**
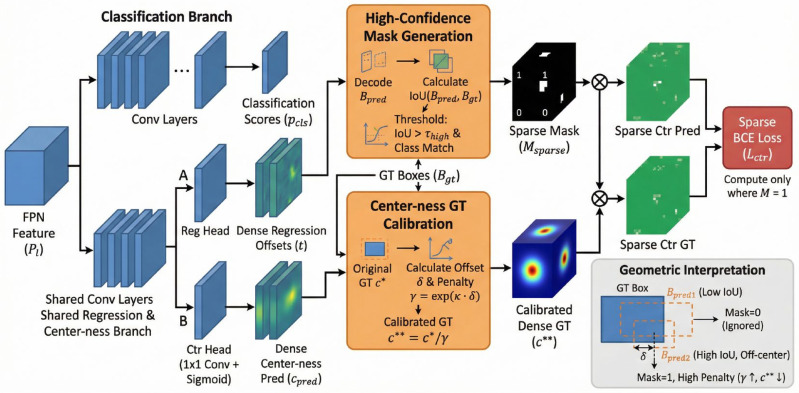
Schematic of the Sparse Center-ness Calibration (SCC) module.

Instead of hand-crafted weighting schemes, we argue that only those pixels whose decoded bounding boxes achieve high overlap with the ground truth should be trusted for learning center-ness. To this end, we propose Sparse Center-ness Calibration (SCC), which enforces sparse, high-confidence supervision on well-aligned predictions. The schematic of the proposed SCC module is illustrated in Figure 4.

Let {P2,P3,P4,P5,P6,P7} denote the feature maps from the FPN. For each spatial location (x,y) at level *l*, FCOS predicts a 4D offset vector $t=\left(l,t,r,b\right)\in R^{4}$ and a class score $p_{\mathrm{cls}}\in R^{C}$. SCC constructs a binary mask $M\in {\left\{0,1\right\}}^{H_{l}\times W_{l}}$ during both training and inference, and performs center-ness supervision only on the subset where M=1.

High-Confidence Mask Generation: During training, let $P_{0}=\left\{\left(x,y\right)|\left(x,y\right)\in B_{\mathrm{gt}}\right\}$ be the initial set of positive samples. For each $\left(x,y\right)\in P_{0}$, decode the predicted box:

(6) $$B_{\mathrm{pred}}\left(x,y\right)=\left[x-l,\;y-t,\;x+r,\;y+b\right],$$

and compute its IoU with the matched ground-truth box:

(7) $$\mathrm{IoU}\left(x,y\right)=\frac{|B_{\mathrm{pred}}\cap B_{\mathrm{gt}}|}{|B_{\mathrm{pred}}\cup B_{\mathrm{gt}}|}.$$

We retain only those locations satisfying $\mathrm{IoU}\left(x,y\right)>\tau_{\mathrm{high}}$ and having a correct classification prediction, where $\tau_{\mathrm{high}}$ represents the high-confidence Intersection over Union (IoU) threshold used to explicitly select high-quality positive samples (set to 0.5 by default):

(8) $$M_{\mathrm{sparse}}\left(x,y\right)=I\left[\mathrm{IoU}\left(x,y\right)>\tau_{\mathrm{high}}\right]\cdot I\left[c\left(x,y\right)=c_{\mathrm{gt}}\right],$$

where I[·] is the indicator function.

Sparse Center-ness Target Refinement: Only for (x,y)∈S={(x,y)∣M(x,y)=1}, we refine the center-ness target. Let $\left(l^{*},t^{*},r^{*},b^{*}\right)$ be the distances from (x,y) to the four sides of the matched GT box. The original center-ness is

(9) $$c^{*}\left(x,y\right)=\sqrt{\frac{\min(l^{*},r^{*})}{\max(l^{*},r^{*})}\cdot \frac{\min(t^{*},b^{*})}{\max(t^{*},b^{*})}}.$$

We define a geometric offset measure:

(10) $$\delta \left(x,y\right)=\max\left(\frac{|l^{*}-r^{*}|}{l^{*}+r^{*}},\;\frac{|t^{*}-b^{*}|}{t^{*}+b^{*}}\right),$$

and we apply an exponential penalty to obtain the calibrated target:

(11) γ(x,y)=exp(κ·δ(x,y))

where κ is a tunable exponential penalty coefficient that actively controls the suppression rate of off-center predictions (empirically set to κ=4 in our experiments). The calibrated target is then formally defined as follows:

(12) $$c^{**}\left(x,y\right)=\frac{c^{*}\left(x,y\right)}{\gamma (x,y)}.$$

This penalizes predictions deviating from the geometric center, effectively suppressing off-center responses.

Sparse Center-ness Head: The SCC head consists of a single 1×1 convolution followed by a Sigmoid activation, sharing hidden layers with the regression branch. The center-ness prediction $c_{\mathrm{pred}}\left(x,y\right)\in \left[0,1\right]$ is trained using binary cross-entropy loss only on S:

(13) $$\begin{array}{c} L_{\mathrm{ctr}}=-\frac{1}{\left|S\right|}\sum_{(x,y)\in S}w\left(x,y\right)\left[c^{**}\left(x,y\right)\log c_{\mathrm{pred}}\left(x,y\right)+\left(1-c^{**}\left(x,y\right)\right)\log\left(1-c_{\mathrm{pred}}\left(x,y\right)\right)\right], \end{array}$$

where w(x,y) serves as a scale-aware weight function that assigns higher loss penalties to smaller objects to emphasize small-object sensitivity. Specifically, w(x,y)=2 if the area of the corresponding ground-truth box $A_{\mathrm{gt}}<32^{2}$, and w(x,y)=1 otherwise.

At inference time, the same head produces a sparse center-ness score $s_{\mathrm{sparse}}\left(x,y\right)$ over the inferred mask $M_{\mathrm{infer}}$, and the final confidence is computed as the geometric mean:

(14) $$s_{\mathrm{final}}\left(x,y\right)=\sqrt{p_{\mathrm{cls}}\left(x,y\right)\cdot s_{\mathrm{sparse}}\left(x,y\right)},$$

followed by standard Non-Maximum Suppression (NMS).

By combining semantic-guided feature fusion and sparse quality calibration, our framework enhances both detection sensitivity and localization reliability—critical capabilities for deploying AI-driven, in-line defect detection systems on edge-ready Automated Optical Inspection (AOI) platforms in semiconductor testing and metrology.

## 4. Experiments

### 4.1. Implementation Details

In this study, we adopt ResNet-50 as the primary backbone network to extract coarse-grained features for our standard baseline models. To address the computational constraints of in-line inspection, we substitute this with the lightweight DLA-34 backbone for our real-time variant.

For the standard models, we follow the widely used “1×” training schedule: the model is trained for 12 epochs with an initial learning rate of 0.01, which is reduced by a factor of 10 at the 8th and 11th epochs. Conversely, for the lightweight model, we adopt an extended training schedule of 360K iterations with multi-scale data augmentation to compensate for the reduced input resolution. Optimization is performed using stochastic gradient descent (SGD) with a momentum of 0.9 and a weight decay of $1\times 10^{-4}$. Training is conducted on 8 GPUs with a batch size of 2 per GPU, resulting in a total batch size of 16.

All models are implemented in PyTorch 2.0.0 and initially trained on the MS COCO train2017 benchmark to establish baseline performance and feature representations. During standard training, input images are resized to 800×1088. For the proposed Sparse Center-ness Calibration (SCC) module, the high-confidence threshold $\tau_{\mathrm{high}}$ is set to 0.5 and the exponential penalty coefficient κ to 4, as determined by our sensitivity analysis.

To ensure robust feature extraction before tackling the highly imbalanced and noisy PCB domain, all models were initially pre-trained on the comprehensive MS COCO dataset. Subsequently, the models were fully fine-tuned on the domain-specific DeepPCB dataset. Following the standard evaluation protocol, the 1500 DeepPCB samples were strictly divided into a training set of 1000 images and a testing set of 500 images. The fine-tuning process was conducted for 24 epochs using Stochastic Gradient Descent (SGD) with a momentum of 0.9. The initial learning rate was set to 0.001 and was decayed by a factor of 10 at the 16th and 22nd epochs. Furthermore, to prevent overfitting and enhance model robustness without altering the static dataset size, on-the-fly data augmentation strategies—specifically random horizontal flipping and photometric distortion—were applied to the training batches.

### 4.2. Evaluation Metrics

To evaluate the detection performance of our proposed method, we employed the standard MS COCO evaluation metrics, including overall Average Precision (AP), AP_50_, and AP_75_. For the evaluation on the domain-specific DeepPCB dataset, we reported the mean Average Precision at an IoU threshold of 0.5 (mAP@0.5) alongside the class-specific AP and Recall for detailed micro-defect categories.

Furthermore, to rigorously assess the practical deployment capability for in-line Automated Optical Inspection (AOI) equipment, we evaluated both the inference speed and model complexity. Model complexity is quantified using the number of parameters (Params in Millions), floating-point operations (GFLOPs), and the physical model weight size (MB). To ensure a strictly fair comparison with existing baselines, all inference speeds (measured in frames per second, FPS) were evaluated on a single NVIDIA GTX 1080Ti GPU. During the latency tests, the input resolution was maintained at 800×1088 for standard models and 512×736 for real-time lightweight variants. All speed measurements were systematically conducted using a batch size of 1 and a Non-Maximum Suppression (NMS) threshold of 0.6, without employing additional post-training acceleration frameworks (e.g., TensorRT). This rigorous setting strictly reflects the real-world end-to-end processing times required in semiconductor manufacturing lines.

### 4.3. Domain-Specific Evaluation on DeepPCB Dataset

To evaluate the generalization and practical value of our framework in real-world semiconductor testing and metrology, we transfer our pre-trained model to the DeepPCB dataset [39]. DeepPCB is a widely recognized benchmark for bare PCB defect detection, containing 1500 image pairs with annotated micro-defects across six common categories: open circuits, shorts, mouse bites, spurs, pinholes, and copper debris. Detecting these flaws is notoriously difficult due to their extremely small scale and the highly complex, repetitive background of circuit traces.

Table 1 compares our method against several state-of-the-art detectors under identical settings.

As shown in Table 1, existing general models struggle to maintain high precision due to the severe semantic noise introduced by the complex PCB background. Nevertheless, our method effectively suppresses false positives and achieves the best overall performance with an mAP@0.5 of 98.7%. Crucially, it outperforms not only popular general-purpose detectors like YOLOv8s (by 0.9%) and the highly competitive RT-DETR-r18 model, but also recently proposed domain-specific architectures designed explicitly for industrial defect detection, such as TCPNet (98.5%) and YOLO-DTS (98.0%).

Furthermore, our approach demonstrates exceptional accuracy across specific defect categories, notably securing the highest AP in open circuits (99.2%) and tying for the highest in mouse bites (98.8%). While specialized networks like YOLO-DTS and TCPNet exhibit slight advantages in specific categories like shorts or spurs, our framework maintains highly competitive and balanced metrics across all six micro-defect types without relying on heavy parallel branches. This demonstrates the strong robustness and comprehensive generalization capability of the SGU and SCC modules in handling challenging micro-defect detection tasks where background interference is substantial.

**Figure 5:**
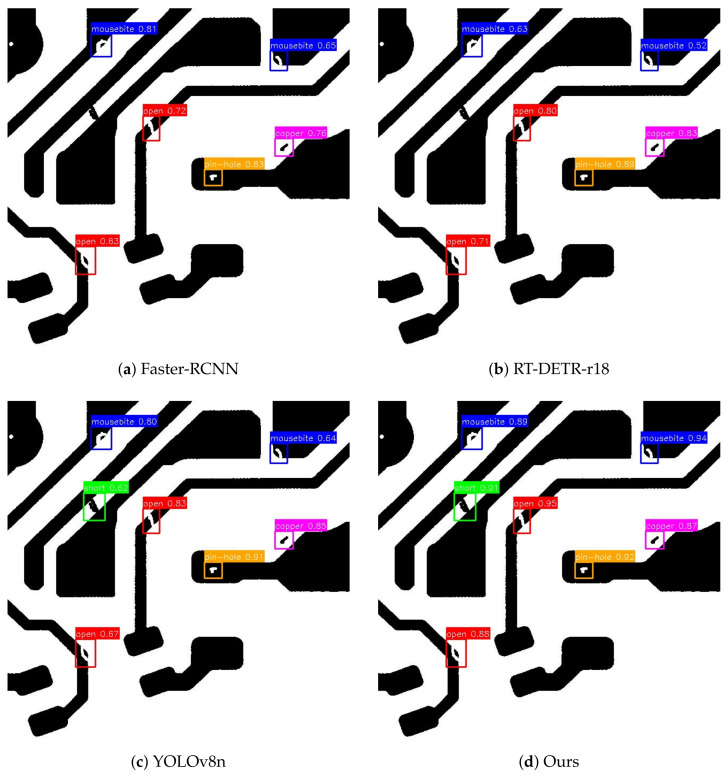
Qualitative comparison on the DeepPCB dataset: (**a**) Faster-RCNN, (**b**) RT-DETR-r18, (**c**) YOLOv8n, and (**d**) Ours.

To qualitatively illustrate the superiority of our approach, Figure 5 visualizes the detection results of different models on typical challenging PCB samples. Compared to baseline methods and popular detectors like YOLOv8s, which frequently exhibit missed detections on extremely tiny defects (e.g., pinholes) and false alarms triggered by dense circuit corners, our proposed PSE-FPN + SCC framework demonstrates exceptional spatial focus. By effectively calibrating the center-ness quality and enhancing shallow semantic features, our method precisely localizes micro-defects while remaining highly robust against background noise.

These results confirm that our framework not only advances foundational object detection performance but also provides a highly effective, AI-driven method for data analysis in semiconductor testing and characterization. By balancing high-precision defect localization with computational efficiency, our approach is ideally suited as an in-line and real-time monitoring method for process control on edge-ready AOI equipment, thereby supporting the scalable advancement of the semiconductor manufacturing industry.

### 4.4. Generalization Evaluation on MS COCO Benchmark

Having established the superior performance of our framework in the specific industrial domain, we further evaluate its generalizability and fundamental detection capabilities by comparing it with state-of-the-art anchor-free detectors on the comprehensive MS COCO benchmark.

As shown in Table 2, the proposed PSE-FPN + SCC framework consistently and significantly improves overall detection accuracy and stringent localization performance among existing anchor-free detectors on COCO test-dev2017 while maintaining real-time inference. Compared with the FCOS baseline, our method raises AP from 38.6% to 41.8%, yielding an absolute gain of 3.2 percentage points. Examining the strict AP_75_ metric reveals that the proposed approach achieves 45.2%, surpassing FCOS by 3.8 percentage points and outperforming GFL and VFL by 2.2 and 1.2 percentage points, respectively. It is worth noting that these gains are obtained without resorting to complicated label assignment or heavy Transformer architectures; instead, they originate from the Semantic-Guided Upsampling Unit and the Sparse Center-ness Calibration, which jointly enhance spatial detail and semantic coherence within the high-resolution P2 layer. Consequently, PSE-FPN + SCC delivers superior overall performance relative to the baseline and contemporary competitors, offering a concise yet effective feature-extraction foundation for subsequent micro-defect detection in resource-constrained metrology scenarios.

### 4.5. Ablation Study

To thoroughly understand the sources of the performance gains observed in both the DeepPCB and COCO comparisons, we conduct systematic ablation studies on both the general MS COCO val2017 dataset and the domain-specific DeepPCB dataset. This dual-evaluation approach allows us to isolate the individual contributions of our proposed modules and validate their effectiveness in both general object detection and specialized industrial inspection scenarios.

#### 4.5.1. Ablation Study on MS COCO Dataset

To systematically validate the effectiveness of each component, we conduct ablation experiments on the COCO val2017 dataset, starting from the baseline FCOS with ResNet-50-FPN. We incrementally introduce three key components: (i) PSE-FPN with only P2-level extension, (ii) full PSE-FPN enhanced with SGU, and (iii) SCC module. Results are summarized in Table 3.

From configurations (1) to (3), we observe that extending the feature pyramid to include P2 (stride = 4) improves AP to 39.1% and AP_50_ to 58.1%, confirming that higher-resolution features preserve spatial structures that are critical for detecting micro-defects. Further integrating the Semantic-Guided Upsampling Unit (SGU) boosts AP to 40.5% and AP_75_ to 43.5%, demonstrating its ability to bridge the semantic gap between deep context and shallow texture via channel–spatial reweighting.

Next, adding SCC alone increases AP to 39.8% and AP_75_ to 43.1%, validating its role in enhancing spatial focusing through sparse, high-confidence supervision. Finally, combining PSE-FPN and SCC achieves the best result—41.8% AP, 60.2% AP_50_, and 45.2% AP_75_—with a sustained real-time speed of 28 FPS, highlighting their complementary benefits.

We further analyze the sensitivity of SCC’s hyperparameters $\tau_{\mathrm{high}}$ and κ, with results shown in Table 4 and Table 5. The optimal configuration occurs at $\tau_{\mathrm{high}}=0.5$ and κ=4, where sufficient high-quality samples are selected while effectively suppressing off-center predictions. Deviations in either direction reduce stability and degrade performance, aligning with the “better fewer but better” principle essential for precise localization of tiny targets.

Theoretically, these optimal values are deeply rooted in the geometric sensitivity of small objects and gradient optimization. For $\tau_{\mathrm{high}}$, minor regression shifts in small objects cause rapid Intersection over Union (IoU) decay. Setting a stringent threshold (≥0.7) leads to positive sample starvation, whereas values below 0.5 introduce severe background noise; thus, 0.5 serves as the optimal statistical balance. For the penalty coefficient, κ=4 generates a steep spatial decay that strictly suppresses off-center false positives in dense COCO scenarios. Higher values (κ≥6), while theoretically sharper, risk gradient vanishing during backpropagation, stalling the network’s convergence.

#### 4.5.2. Ablation Study on DeepPCB Dataset

To robustly validate the specific effectiveness of the proposed modules for our intended industrial domain, we further conducted comprehensive ablation studies on the DeepPCB dataset. The baseline model is the standard FCOS architecture equipped with a lightweight backbone. We progressively integrated the Semantic-Guided Upsampling Unit (SGU), the Progressive Semantic Enhancement Feature Pyramid Network (PSE-FPN), and the Sparse Center-ness Calibration (SCC) module. The results are summarized in Table 6.

As shown in Table 6, the baseline FCOS model achieves an mAP@0.5 of 95.8% and struggles significantly with the severe semantic noise introduced by the complex PCB background. The integration of the SGU module yields a notable improvement of 1.1% in mAP@0.5, indicating that recovering semantic information during the upsampling phase is highly beneficial for locating subtle defect boundaries. Furthermore, replacing the standard FPN with our PSE-FPN boosts the overall mAP@0.5 to 97.8% (a 0.9% increase). This confirms that maintaining high-resolution semantic consistency across the progressive feature pyramid effectively mitigates the feature dilution of micro-defects.

Finally, the addition of the SCC module brings the mAP@0.5 to our state-of-the-art result of 98.7%. The SCC module proves particularly effective in the DeepPCB dataset, where complex and repetitive circuit textures often cause severe false positives. By sparsely calibrating the center-ness scores, the SCC successfully suppresses low-quality predictions in the background, significantly enhancing precision and enabling highly reliable micro-defect inspection in industrial scenarios.

### 4.6. Real-Time and Lightweight Models for In-Line Defect Inspection

While the standard ResNet-50-based framework demonstrates exceptional accuracy and mechanism soundness, deploying AI-driven metrology models directly onto edge devices integrated within semiconductor equipment often faces strict computational and thermal constraints. To bridge the gap between theoretical performance and in-line industrial deployment, we further evaluate a real-time and lightweight version of our proposed algorithm.

In the context of advanced semiconductor manufacturing, in-line inspection and real-time defect monitoring are critical for process control and yield improvement. However, deploying AI-driven metrology models directly onto edge devices integrated within semiconductor equipment often faces strict computational and thermal constraints. To address the need for high-throughput Automated Optical Inspection (AOI), we further develop a real-time and lightweight version of our proposed algorithm.

Following the real-time settings of the baseline FCOS [27], we introduce several structural and training modifications. First, to significantly decrease inference latency, we reduce the shorter side of the input images from 800 to 512 and the maximum longer side from 1333 to 736. With this reduced input size, higher-level FPN features ($P_{6}$ and $P_{7}$) become less critical for nanoscale or microscale semiconductor defects and are therefore removed to further accelerate inference. Second, we adopt a more aggressive training strategy to compensate for the resolution reduction. Specifically, we use multi-scale data augmentation where the shorter side is randomly sampled from 256 to 608. We also utilize Synchronized Batch Normalization (SyncBN) and extend the training schedule to 360 K iterations. The learning rate is decayed by a factor of 10 at 300 K and 340 K iterations.

For the backbone network, we replace the heavy ResNet-50 with the lightweight DLA-34. To push the speed further for extreme high-speed production lines, we also evaluate a variant that shares the convolutional towers between the classification and regression branches.

Table 7 presents the speed and accuracy trade-off. While the baseline FCOS-RT (DLA-34) achieves 40.3% AP at 46 FPS, our lightweight variant (DLA-34) achieves a superior 41.6% AP at a highly competitive speed of 42 FPS. Remarkably, in high-speed real-time AOI settings with reduced input resolution, the semantic gap and detail loss for tiny defects are usually exacerbated. However, our PSE-FPN module inherently mitigates this by retaining the high-resolution $P_{2}$ level and utilizing the Semantic-Guided Upsampling Unit (SGU). Combined with the Sparse Center-ness Calibration (SCC) module, the lightweight variant effectively suppresses low-quality background noises (e.g., complex circuit patterns or periodic wafer surface variations) that frequently interfere with defect identification.

Even when sharing the towers to maximize inference speed (reaching 50 FPS), our model still maintains a high accuracy of 40.5% AP, outperforming the baseline FCOS-RT with shared towers (39.1% AP) by a significant margin. This demonstrates that our proposed semantic-guided architecture is not only highly accurate but also efficient enough to serve as a robust, real-time data analysis method for advanced semiconductor testing and metrology.

## 5. Discussion

While the proposed semantic-enhanced FCOS framework demonstrates superior performance on standard benchmarks and PCB defect datasets, it is essential to discuss its generalization capabilities and current limitations in broader industrial contexts.

Generalization to Other Semiconductor Domains: The architectural improvements introduced in this study—specifically the Semantic-Guided Upsampling Unit (SGU) and Sparse Center-ness Calibration (SCC)—are fundamentally task-agnostic. Because they focus on general feature pyramid semantic alignment and quality estimation calibration rather than PCB-specific heuristics, our framework possesses strong potential for generalization. It can be readily adapted to other critical semiconductor manufacturing tasks, such as in-line wafer defect inspection (e.g., detecting surface scratches, particle contaminations, and crystalline defects on bare silicon wafers) and IC packaging surface anomalies, provided that appropriate domain-specific transfer learning is applied.

Limitations and Future Work: Despite these architectural advantages, the current framework faces certain challenges when deployed in extreme industrial environments. First, for exceptionally tiny micro-defects, spatial features may still be severely diluted during the backbone’s cascaded downsampling process, even with the introduction of our high-resolution P2 level. Second, in high-noise industrial images characterized by uneven illumination or severe texture interference, the initial bounding box predictions may degrade. This degradation can subsequently affect the efficacy of the SCC module, as it relies on relatively accurate initial localization to generate the high-confidence calibration mask.

Future work will focus on addressing these limitations by exploring sub-pixel feature enhancement techniques and frequency-domain noise suppression modules, thereby further improving the robustness and sensitivity of extremely small-object detection in harsh optical environments.

## 6. Conclusions

This study addresses the critical challenge of yield management and quality control in semiconductor manufacturing. To advance AI-driven in-line defect detection, we propose a semantic-enhanced FCOS framework. This framework is specifically tailored to detect extremely small and sparse micro-defects—such as mouse bites, pinholes, and open circuits—on bare Printed Circuit Boards (PCBs) within Automated Optical Inspection (AOI) systems.

Our method introduces two synergistic components: the Semantic-Guided Upsampling Unit (SGU) and the Sparse Center-ness Calibration (SCC) module. Together, they mitigate two primary limitations of current anchor-free detectors: semantic misalignment during feature fusion and inefficient quality estimation caused by dense supervision. Specifically, the SGU is integrated into a Progressive Semantic-Enhanced Feature Pyramid Network (PSE-FPN). This progressive network extends multi-scale representations to the high-resolution P2 level, effectively strengthening the bottom-up semantic flow. Simultaneously, the SCC module is embedded directly into the detection head to provide sparse, high-confidence localization supervision.

Extensive experiments validate the effectiveness of our design. On the general MS COCO benchmark, the proposed model achieves 41.8% AP at a real-time speed of 28 FPS on a single NVIDIA 1080Ti GPU. To satisfy the strict throughput requirements of production lines, our lightweight variant further accelerates inference to 42 FPS while maintaining a highly competitive 41.6% AP.

For real-world semiconductor metrology, application validation on the DeepPCB dataset yields a superior mAP@0.5 of 98.7%. Our method significantly outperforms contemporary detectors, such as YOLOv8s and RT-DETR-r18. This performance confirms the framework’s strong generalization ability and its robustness against the complex, repetitive backgrounds of circuit traces. Notably, these gains are achieved without relying on heavy Transformer architectures or complex label assignment strategies.

Ultimately, this work demonstrates that semantics-aware, lightweight architectures can effectively balance high precision with computational efficiency. By co-designing feature fusion and prediction calibration mechanisms tailored for micro-defects, our framework provides a scalable, intelligent solution for real-time quality assurance in next-generation semiconductor manufacturing.

## Figures and Tables

**Table 1:** DeepPCB comparison test.

Methods	mAP@0.5/%	AP/%					
		Open	Short	Mousebite	Spur	Copper	Pin-Hole
Faster-RCNN	97.5	96.8	95.4	97.8	98.7	98.9	97.4
RT-DETR-r18	98.6	98.5	97.7	98.1	98.5	99.5	99.3
SSD	95.9	93.1	94.5	95.7	96.7	96.9	98.7
YOLOv5s	97.2	97.1	96.1	98.1	96.1	98.3	97.5
YOLOX	97.5	95.9	97.3	97.1	97.8	97.8	98.8
YOLOv7-tiny	93.5	96.3	85.7	93.9	94.6	96.1	94.3
YOLOv8n	95.7	96.8	90.6	98.0	95.8	96.4	96.7
YOLOv8s	97.8	98.2	95.0	98.8	97.5	98.4	98.6
YOLOv12s	98.3	98.6	96.5	98.4	97.9	99.0	99.1
TCPNet	98.5	98.8	98.7	98.3	98.5	98.4	98.3
YOLO-DTS	98.0	95.4	98.9	97.7	98.7	98.3	99.2
Ours	98.7	99.2	97.8	98.8	98.4	99.3	98.9

**Table 2:** Performance comparison on COCO test-dev2017.

Method	AP	AP_50_	AP_75_	Reference
FoveaBox [40]	36.4	55.8	38.8	-
FCOS [27]	38.6	57.4	41.4	ICCV
ATSS [36]	39.2	57.4	42.4	CVPR
PAA [41]	40.4	58.4	44.3	ECCV
OTA [42]	40.7	58.4	44.3	CVPR
GFL [43]	39.9	58.5	43.0	NeurIPS
VFL [44]	40.2	58.2	44.0	CVPR
FCOS + GFLv2	40.6	58.2	43.9	CVPR
ATSS + GFLv2	41.1	58.8	44.9	CVPR
Musu [45]	40.6	58.9	44.3	ICCV
TOOD [46]	40.3	58.5	43.8	ICCV
DW [47]	41.5	59.8	45.0	CVPR
MFIL-FCOS [35]	41.6	59.9	45.0	-
Ours	41.8	60.2	45.2	-

**Table 3:** Ablation study of proposed modules on COCO val2017.

Baseline (FCOS)	P2-Extension	SGU	SCC	AP (%)	AP_50_ (%)	AP_75_ (%)
✓				38.6	57.4	41.4
✓	✓			39.1	58.1	42.0
✓			✓	39.8	58.7	43.1
✓	✓	✓		40.5	59.4	43.5
✓	✓	✓	✓	41.8	60.2	45.2

**Table 4:** Hyperparameter sensitivity analysis of the high-confidence IoU threshold $\tau_{\mathrm{high}}$ in the SCC module on COCO val2017.

$\tau_{\mathrm{high}}$	AP	AP_50_	AP_75_
0.4	41.0	60.2	43.9
0.5	41.8	60.2	45.2
0.6	41.3	60.0	44.5
0.7	40.6	59.4	43.8

**Table 5:** Hyperparameter sensitivity analysis of the exponential penalty coefficient κ in the SCC module on COCO val2017.

κ	AP	AP_50_	AP_75_
2	41.2	60.1	44.9
4	41.8	60.2	45.2
6	41.4	59.8	44.5
8	40.8	59.4	43.8

**Table 6:** Ablation study of individual modules on the DeepPCB dataset. The baseline is the standard FCOS.

Model	SGU	PSE-FPN	SCC	mAP@0.5 (%)
Baseline				95.8
Variant 1	✓			96.9
Variant 2	✓	✓		97.8
Ours	✓	✓	✓	98.7

**Table 7:** Performance and complexity of real-time and lightweight models on COCO val2017. FPS is measured on a single NVIDIA 1080Ti GPU.

Method	Backbone	Params (M)	GFLOPs	Size (MB)	FPS	AP (%)	AP_50_ (%)
FCOS-RT	DLA-34	19.5	45.0	78.0	46	40.3	59.1
FCOS-RT (shared towers)	DLA-34	15.2	38.0	61.0	52	39.1	58.3
Ours-RT	DLA-34	20.1	58.0	80.5	42	41.6	59.6
Ours-RT (shared towers)	DLA-34	15.8	51.0	63.5	50	40.5	59.1

## Data Availability

The data presented in this study are available on request from the corresponding author.
